# Contextual Factors and Decision-Making in the Behavior of Finalization in the Positional Attack in Beach Handball: Differences by Gender Through Polar Coordinates Analysis

**DOI:** 10.3389/fpsyg.2019.01386

**Published:** 2019-06-13

**Authors:** Juan A. Vázquez-Diz, Juan P. Morillo-Baro, Rafael E. Reigal, Verónica Morales-Sánchez, Antonio Hernández-Mendo

**Affiliations:** Departamento Psicología Social, Trabajo Social, Antropología Social y Estudios de Asia Oriental, Universidad de Málaga, Málaga, Spain

**Keywords:** mixed methods, systematic observation, polar coordinates, beach handball, decision making

## Abstract

The aim of this study was to analyze, in the framework of the mixed methods, the relationship of different contextual factors with the decisions shown in the finalization of the positional attack in beach handball. For this reason, a polar coordinates analysis was carried out, by gender, using as focal behaviors the simple and double goal, and the loss of possession of the attacking team, which are considered decisive in assessing the success of the decisions at the end of the positional attack. These focal behaviors have been linked to criteria that characterize the attack situation such as the minute, the score, the numerical balance, the defensive system and the duration. A total of 24 sessions were observed with the HOISAN computer software, using an *ad hoc* designed tool. The model of observation used was punctual, multidimensional, and nomothetic. The observation unit used for the positional attack from when the specialist gains control of play until the possession changes. The results obtained showed that an advantage of goals scored and the numerical balance situations of the teams modulate significantly the appropriate decision made by both categories. However, they also indicated differences in the flow of pairing behaviors for both categories in some aspects. Specifically, it has been observed that a longer duration of the attack in the female category has been linked significantly to a successful performance and the results also show that an elaborated attack is related to successful behaviors. In the male category, it has been observed that the technical fouls made by the attackers increase in the last minutes of the match. Likewise, as regards the to the opposing team’s defensive system, the results in the male category are related to successful behaviors before any of them; whereas, in the female category, when playing against a defensive pressure system, the results relate more to errors during the performance of the pass and reception. The use of polar coordinates for the estimation of technical-tactical relations allows, from a psychological point of view, determine the techniques and procedures of psychological intervention that optimize the action resources of the players individually and the team as a collective.

## Introduction

Published research on the planning of training in collective sports emphasizes the importance of the gradual increase of physical, technical and tactical demands in the competition in recent decades (Carling, 2011; Sarmento et al., 2017). This fact has led to the need to increase specific knowledge to carry out proper planning and make the preparation of the team as complete as possible (Nunes et al., 2015). Beach handball has also been immersed in this tendency, proof of this is the constant evolution that has developed in all aspects of the game since its beginnings in the nineties to today (Morillo-Baro and Hernández-Mendo, 2015; Navarro et al., 2018), promoting in the professionals of this sport a continuous update and innovation.

Despite being a method that comes from the handball court, it also brings with it many differences, turning it into a completely new method (Belančić, 2005). One of the most significant differences lies in the possibility of scoring goals, each with a different point value, achieved through a variation of types of game play. Goals of simple value are achieved by means of a classic throw made by any of the players except the goalkeeper-specialist, and goals of double value are made by a specialist's shot, throwing from a dive shot or throwing in 360 degrees (Ostoić and Ohnjec, 2015). The commitment of the International Handball Federation (IHF) and the European Handball Federation (EHF) by this modality is evident. This has led an increase in the number of international tournaments that are held every year, such as world championships and continental championships (Morillo-Baro et al., 2015).

Being a collective sport, beach handball has an open character. This means that it presents a high degree of uncertainty, which can lead to thinking about randomness of the game (Jiménez-Salas and Hernández-Mendo, 2016). So, to compete at the highest level it requires a high level of selective attention that allows the athlete to capture the most important stimuli of the game for further processing, enabling a correct decision making at all times (Gil-Arias et al., 2016). During the game there are constant changes of players and different situations of numerical superiority or inferiority that occur at a high speed, this recreates a continuously changing environment, where athletes have to make decisions constantly to adapt to the large number of different game situations found (Gil-Arias et al., 2012).

There are numerous factors or situational variables that can alter the decisions made during the course of a match, such as the type or level of the opponent he/she faces to, the result, the moment of the game or the evolution of the score (Aguilar et al., 2012). Another aspect is the possibility that a player from the opposing team receives a suspension, creating a situation of double numerical superiority, creating a different decision situation that can lead to a change in the decisional performance of the attacking team (Aguilar et al., 2012). There is research that has studied the influence of these determining factors in athletes’ decisions. In handball, there has been studies concerning the situations of numerical inequality (Aguilar et al., 2012). Research in volleyball analyzes the relationship between decision-making and performance in three actions of the game (reception, stage, attack) (Conejero et al., 2018). Studies in soccer show the results of the influence of contextual factors on the decision making of highly qualified players through semi-structured personal interviews, where it was clear the importance of considering the dynamic and static context in which players make decisions (Levi and Jackson, 2018). The study of these factors or external variables that determine the decision making of athletes is the central axis around which this research has revolved. Araújo et al. (2016) propose to study the decision-making process based on the ecological perspective, observing the results of the athletes’ performance through patterns of success against unsuccessful patterns considering various factors such as the game space, the team mates and rivals. The observation of this result of performance can be done through a Field Format and Systems mixed observation tool of E/ME categories (Exhaustive and Mutually Exclusive) (Araújo et al., 2016).

The investigations carried out in the field of sports, within the framework of the mixed methods, have to connect qualitative and quantitative elements (Plano-Clark and Sanders, 2015). In this way, the Observational Methodology (OM) is a technique and/or methodology suitable for analyzing performance behavior in sport (Anguera and Hernández-Mendo, 2013; Portell et al., 2015). Also, systematic observation is widely applicable and offers an optimal balance between rigor and flexibility. The wide scope of opportunities available for processing data derived from observation that supports the idea that purely observational studies should be considered as mixed methods research studies, even though they constitute a somewhat special case and do not follow traditional patterns (Anguera et al., 2017). Numerous tools have been created to observe and analyze the different situations of play that occur during a handball match (González, 2012; González et al., 2013; Sousa et al., 2014; Loffing et al., 2015; Helm et al., 2016; Jiménez-Salas and Hernández-Mendo, 2016) and beach handball (Morillo-Baro and Hernández-Mendo, 2015). In recent years, the analysis of decision-making has gained great interest with the publication of different investigations (Martín et al., 2013; Weigel et al., 2015). Specifically, in handball observations of the referees have showed the influence of playing time on the decision making in relation to the tactical means employed (Prudente et al., 2017) and the influence on the decisions depending on the area of the referee’s placement (Morillo-Baro et al., 2017).

The OM takes place in natural or habitual contexts, and is based on a scientific procedure that, depending on the objectives that are stated, expresses the occurrence of perceptible behaviors, for its subsequent registration through an observation tool built specifically using the appropriate parameters. The motor behavior can be studied through the most characteristic analysis of OM, the sequential analysis of delays and the analysis of polar coordinates (Anguera and Hernández-Mendo, 2013, 2014, 2015).

In recent years, the use of polar coordinates analysis as one of the most used analysis techniques has been developed in Sports Sciences (Morillo-Baro et al., 2015; Araújo et al., 2016; Castañer et al., 2016; Maneiro et al., 2019). Numerous investigations have used this analysis of polar coordinates focusing on different sports methods, a great number of which concerning football (Echeazarra et al., 2015; Castañer et al., 2017; Maneiro and Amatria, 2018), but also in other sports such as karate or basketball (Nunes et al., 2015; Riveiro-Bozada et al., 2016). In handball, the study was focused on the offensive situations of two against two, obtaining the different effective behavioral vectors (Sousa et al., 2014). Moreover, specifically, in beach handball the positional attack was studied, determining differences by gender (Morillo-Baro et al., 2015; Navarro et al., 2018).

Based on all this background on beach handball as an object of study, the objective is to study the relationship of some situational factors in the decisions of beach handball players through the analysis of polar coordinate.

## Materials and Methods

For the development of the research, a nomothetic, punctual, and multidimensional design was chosen (Anguera et al., 2011). The sequence of positional attack that has been observed begins from the moment the specialist gains play until the possession is changed, being this, the unit of observation used.

### Participants

Six teams have been observed for each category of the 24 that participated in total during the 2016 Senior Spanish Cup (*M* ± SD: age male = 26.67 ± 5.85; age female = 23.27 ± 5.74). The six teams have been chosen randomly with the intention of examining two observations from each team. There were 24 observation sessions, 12 in the male category and 12 in the female category, these being the number of sessions that were estimated necessary to be able to generalize with precision (Morillo-Baro and Hernández-Mendo, 2015). The analyzed matches are shown in Table 1.

**Table 1:** Analyzed matches.

Category	Matches
**Male**	C. BMP. Alcala – Zonabalonmano Cadiz
	C. BMP. Ciudad de Malaga – C. BMP. Bahia de Algeciras (team no observed)
	C. BMP. Ciudad de Malaga – C. BMP. Barbate “B” (team no observed)
	BHC Plan B Barcelona – C. BMP. Barbate
	BHC Plan B Barcelona – Zonabalonmano Cadiz
	Pinturas Andalucia BMP Sevilla – C. BMP. Barbate
	Pinturas Andalucia BMP Sevilla – C. BMP. Alcala
**Female**	C. BMP. Algeciras – C. BMP. Ciudad de Malaga
	C. BMP. Algeciras – Deporte y Empresa Clinicas Rincon Malaga
	C. BMP. Ciudad de Malaga – C. BMP. Getasur juvenil Madrid
	C. BMP. Getasur junior Madrid – Deporte y Empresa Malaga
	C. BMP. Getasur junior Madrid – C. BMP. Getasur juvenil Madrid
	Jugui SOS Valencia (team no observed) – Deporte y Empresa Málaga
	Grupo Llopis BMP Sevilla (team no observed) – C. BMP. Algeciras

### Instruments

**FIGURE 1:**
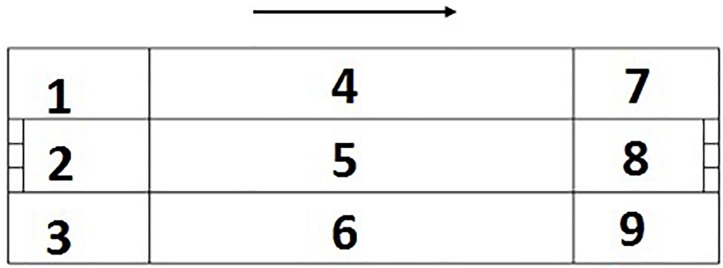
Division of game spaces. The numbering is related to the sense of attack.

The Royal Spanish Handball Federation (RFEBM) was in charge of recording the matches. The HOISAN computer software was used to perform the data recording and coding, the sequential analysis, the polar coordinate analysis and its vectorial representation (Hernández-Mendo et al., 2012). The tool used was already created and was *ad hoc* designed and had passed the quality tests of the data that are required in OM (Morillo-Baro and Hernández-Mendo, 2015). It consists of 11 criteria and 90 categories that correspond chronologically with the development of an attack on beach handball (Morillo-Baro and Hernández-Mendo, 2015). The criteria of the observation tool and the categories that make up each one of them are shown in Table 2. Next, Figure 1 shows the division of games spaces.

**Table 2:** Observation instrument: criteria and corresponding categories and codes.

Criterion	Categories	Criterion	Categories
**1. Minute**	**M1**: minute one of first half. **M2**: minute two of first half. **M3**: minute three of first half. **M4**: minute four of first half. **M5**: minute five of first half. **M6**: minute six of first half. **M7**: minute seven of first half. **M8**: minute eight of first half. **M9**: minute nine of first half. **M10**: minute ten of first half. **M11**: minute one of second half. **M12**: minute two of second half. **M13**: minute three of second half. **M14**: minute four of second half. **M15**: minute five of second half. **M16:** minute six of second half. **M17:** minute seven of second half. **M18:** minute eight of second half. **M19**: minute nine of second half. **M20**: minute ten of second half. **MGOAL1**: golden goal of first half. **MGOAL2**: golden goal of second half.	**2. Score**	**TIE:** tie. **1WIN**: observed team is winning by one point. **2WIN:** observed team is winning by two points. **M2WIN**: observed team is winning by more than two points. **1LOS**: observed team is losing by one point. **2LOS**: observed team is losing by two points. **M2LOS**: observed team is losing by more than two points.
**3. Numerical balance**	**EQUAL:** equality. **1SUP**: advantage of one. **M1SUP**: advantage of more than one. **1INF**: disadvantage of one. **M1INF**: disadvantage of more than one.	**4. Defensive system**	**S30**: 3:0 defensive system. **S21**: 2:1 defensive system. **S2M1**: 2+1 defensive system. **SPRES:** individual defensive system. **SRETR:** retreat defensive system. **S20**: 2:0 defensive system.
**5. Zone of end of attack**	**Z1:** attack ends in zone 1. **Z2:** attack ends in zone 2. **Z3:** attack ends in zone 3. **Z4:** attack ends in zone 4. **Z5:** attack ends in zone 5. **Z6:** attack ends in zone 6. **Z7:** attack ends in zone 7. **Z8:** attack ends in zone 8. **Z9:** attack ends in zone 9.	**6. Substitution area**	**SAYES:** attack ends in one of the zones adjacent to the observed team’s substitution area. **SANO:** attack ends in one of the zones adjacent to the opposing team’s substitution area. **SAMID:** attack ends in one of the central zones.
**7. Assisting player**	**ASPE:** assist by specialist. **ALWING:** assist by left wing. **ARWING:** assist by right wing. **ACENT:** assist by center. **APIV:** assist by pivot. **NASIS:** no assist.	**8. Player who ends attack**	**FSPE:** specialist ends attack **FLWING:** left wing ends attack. **FRWING:** right wing ends attack. **FCENT:** center ends attack. **FPIV:** pivot ends attack.
**9. How attack ends**	**FLY:** attack ends with in-flight shot. **SPIN:** attack ends with spin shot. **SHOT:** attack ends with shot. **6M:** attack ends with 6-m throw. **TURN:** attack ends with passing or catching error. **TECNF:** attack ends with technical foul.	**10. Result of end of attack**	**GOAL1**: one-point goal. **GOAL2**: two-point goal. **MSHOT:** missed shot. **CPOSE:** change of possession. **G1SUSP**: one-point goal and suspension. **G2SUSP**: two-point goal and suspension. **6MGOAL**: goal scored by 6-m throw. **6MM**: missed 6-m throw. **GOAL1R**: one-point rebound goal. **GOAL2R**: two-points rebound goal. **6MGSUSP**: goal scored by 6-m throw and suspension. **6MMSUSP**: missed 6-m throw and suspension. **GOAL1RSUSP**: one-point rebound goal and suspension. **GOAL2RSUSP**: two-point rebound goal and suspension.
**11. Duration**	**D05**: between 0 and 5 s.		
	**D610**: between 6 and 10 s.		
	**D1115**: between 11 and 15 s.		
	**D1620**: between 16 and 20 s.		
	**D2125**: between 21 and 25 s.		
	**D2630**: between 26 and 30 s.		
	**DM30:** more than 30 s.		

### Procedure

#### Data Quality

The correlations have been obtained, once the data has been collected, with the coefficients of Pearson, Spearman, Tau b of Kendall and the coefficient Phi besides the concordance index of Cohen Kappa for the complete session. This is a necessary process in OM since the observer must have the necessary guarantee on the quality of the data obtained (Anguera, 2003). The results are shown in Table 3.

**Table 3:** Intra- and inter-observer agreement.

Coefficient for entire session	Intra-observer agreement (Obs. 1 vs. Obs. 1bis)	Inter-observer agreement (Obs. 1 vs. Obs. 2)
Pearson’s	0.98	0.97
Spearman’s (ρ)	0.95	0.89
Kendall’s tau-b	0.95	0.91
Kappa	0.94	0.91
Phi	0.89	0.85

As it can be observed, the Cohen Kappa index for this research exceeds 0.90 for both intra-observer (0.94) and inter-observer (0.91) reliability. Due to this, it is considered that there is high reliability, according to the values proposed by Gelfand and Hartmann (1975) or Landis and Koch (1977).

#### Generalizability Analysis

The Theory of Generalizability has been applied through the use of SAGT computer software (Hernández-Mendo et al., 2016). A design of two facets, categories and observers = C/O has been determined, which has served to determine the intra-observer and inter-observer concordance. The homogeneity of the categories has also been assessed, also using a two-faceted design, but with an O/C design. In the Theory of Generalizability, the definitions of reliability, validity and precision are unified. The Generalizability study consists basically of four phases: (1) Definition of the facets of study; (2) Analysis of variance of the scores obtained on the study facets; (3) Calculation of the error components; (4) Optimization of the Generalizability coefficients (Blanco-Villaseñor et al., 2014).

To determine inter-observer reliability, a two-faceted design (C = O) was established, as we previously mentioned. The results show that practically all the variability is associated with the facet categories (97.12%), being 0 for the facet observers and of 2.28% for the interaction of the facets categories/observers. The generalizability coefficients for this structure show a result of 0.98, which corresponds to excellent results.

The same design was taken to determine intra-observer reliability. The obtained results showed that almost all the variability was associated to the facet categories (97.99%) being 0 for the facet observers and resulting for the interaction of the facets categories/observers 2.01%. The generalizability coefficients for this design structure showed excellent results when it was obtained a value of 0.99.

Finally, the homogeneity of the categories was assessed, to do it, a two-faceted design (observers and categories = O/C) was chosen, thanks to this design, the degree of differentiation of the proposed categories was obtained. The generalization coefficients obtained for this design are zero (0.00), which means that the homogeneity of the categories is optimal in the sense of differentiators.

#### Polar Coordinate Analysis

Sackett (1980) was the precursor of this technique, which over the years was optimized by Anguera’s (1997) genuine technique, which allows a drastic reduction of data and a graphic representation of the vectors that determine the interrelations between the different categories that make up the proposed taxonomic system (Hernández-Mendo and Anguera, 1999; Gorospe and Anguera, 2000). This technique is supported by a sequential analysis of prospective delays (Sackett, 1980) and retrospective, with the genuine technique (Anguera, 1997) of the successive behaviors that occurred. The values obtained in the calculation of the conditioned probability will allow to obtain the Zsum parameter (Zsum = Σz/√n, where n is the number of delays) (Cochran, 1954). The distribution of this Zsum parameter has one 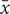 = 0 and one *SD* = 1. The interrelation map of behavior or map of polar coordinates (Gorospe and Anguera, 2000) is obtained from obtaining these values. It is necessary to determine the value of the vectors (they must be equal or greater than 1.96 to be considered significant) for the preparation of behavioral maps. The module or length of the radius is obtained by taking the square root of the sum of the square of the Zsum of the X (prospective) and the square of the Zsum of the Y (retrospective). The angle of the vector (φ) (which will depend on the quadrant where it is located) marking the nature of the relationship (Castellano and Hernández-Mendo, 2003). This angle (φ) is calculated as φ = Arc sine of Y/Radius.

The HOISAN computer program (Hernández-Mendo et al., 2012) allowed the coding and analysis of polar coordinates. Firstly, once the criterion behavior was selected, a sequential analysis was performed for each category of all the observations, obtaining the Z results for a range of delays −5 and 5. Once these values were obtained, the necessary calculations were made to obtain the parameters Zsum (prospective and retrospective), the assignment of the quadrant, the module, the angle and the transformed angle (AT) for the rest of the categories (Castellano and Hernández-Mendo, 2003):

Quadrant I [+, +]: The criterion behavior is excited with respect to the pairing behavior in retrospective and prospective perspective.

Quadrant II [−, +]: The criterion behavior has a relation with respect to the pairing of excitation in retrospective perspective and of inhibition in prospective perspective.

Quadrant III [−, −]: The criterion behavior has a relation with respect to the pairing of inhibition in retrospective and prospective perspective.

Quadrant IV [+,−]: The criterion behavior has a relationship with the behavior of arousal pairing in prospective perspective and inhibition in retrospective perspective.

The behaviors selected as criteria (focal) have been:

- GOAL2: this behavior is what refers to the final objective of the attack and, therefore, its appearance can be associated with a pattern of success in the decision made by the attacking team. In addition, it includes all the possibilities of scoring a goal of double value (flight, turn, throw of the goalkeeper and throw of 6 m).
- TURN and TECNF: both behaviors refer to the loss of possession of the ball by the team that attacks either by an own error or a forced error by the defensive action of the opposing team. Therefore, both are associated with an unsuccessful pattern in the decision made by the attacking team.

It has been decided to link these behaviors only with the criteria of the observation tool that are related to external factors or variables and that can modulate the decision making of the athletes: Minute, Score, Numerical Balance, Defensive System, and Duration.

## Results

Then, it can observe the results obtained by analyzing polar coordinates for the selected behaviors as a criterion. Table 4 shows the pairing behaviors that are significantly linked when the criterion behavior is GOAL2 (per quadrant and category).

**Table 4:** Relationships between focal behavior GOAL2 and conditional behaviors.

Criterion behavior	Q	Male			Female		
		Pairing behavior	Vector module	T.A.	Pairing behavior	Vector module	T.A.
**GOAL2**	I	M2WIN	7.63	10.76	M2WIN	4.14	2.43
		1LOS	3.42	59.02	2WIN	3.90	8.50
		SRETR	3.05	35.18	S30	2.86	64.26
		M15	3.00	81.84			
	II	M6	3.00	137.12	2LOS	2.32	168.94
		M1	2.84	139.71			
		M7	2.72	159.41			
	III	M2LOS	7.38	201.54	M2LOS	5.70	194.79
		S21	2.34	219.78	S20	2.09	216.02
	IV	2WIN	2.63	305.16	D1115	1.98	346.23
		M18	2.50	346.10			

*Q, quadrant; T.A., transformed angle; GOAL2, two-points goal; M2WIN, observed team is winning by more than two points; 1WIN, observed team is winning by one point; SRETR, individual defensive system; M15, minute five of second half; M6, minute six of first half; M1, minute one of first half; M7, minute seven of first half; M2LOS, observed team is losing by more than two points; S21, 2:1 defensive system; 2WIN, observed team is winning by two points; M18, minute eight of second half; S30, 3:0 defensive system; 2LOS, observed team is losing by two points; S20, 2:0 defensive system; D1115, between 11 and 15 s.*

In quadrant I, where it is excited with respect to pairing behavior in retrospective and prospective perspective. In masculine category, it is associated with four pairing behaviors: the score of more than two goals in favor (M2WIN), the score of a goal in favor (1WIN), the defensive system of the opposing team (SRETR) and the minute fifteen of the second half (M15). While in the female category, it is associated with three pairing behaviors: the score of more than two goals in favor (M2WIN), the score of two goals in favor (2WIN) and the defensive system of the opposing team 3: 0 (S30). It emphasizes that in both categories the two behaviors that more intensity present in their relation refers to the score in favor of the attacking team.

In quadrant II, where the criterion behavior has a relationship with respect to the mate of excitation in retrospective perspective and inhibition in prospective perspective, in the male category, three pairing behaviors that belong to the same criterion, minute number, are excited, the minute one, six, and seven of the first half (M1, M6, and M7). Meanwhile, in the female category, there is only a pairing behavior that is the score of two goals against (2LOS).

In quadrant III, the criterion behavior has a relationship with respect to the pairing of inhibition in retrospective and prospective perspective. In both categories, two pairing behaviors are excited, and for both of them, the greater radius presents the score of more than two goals against (M2LOS).

Finally, in quadrant IV, the criterion behavior has a relationship with the behavior of excitation matching in prospective perspective and inhibition in retrospective perspective. In male category, it is associated with two behaviors of pairing: the score of two goals in favor (2WIN) and the minute eight of the second half (M18); whereas for feminine category only one appears, the duration of the attack between eleven and fifteen seconds (D1115).

**FIGURE 2:**
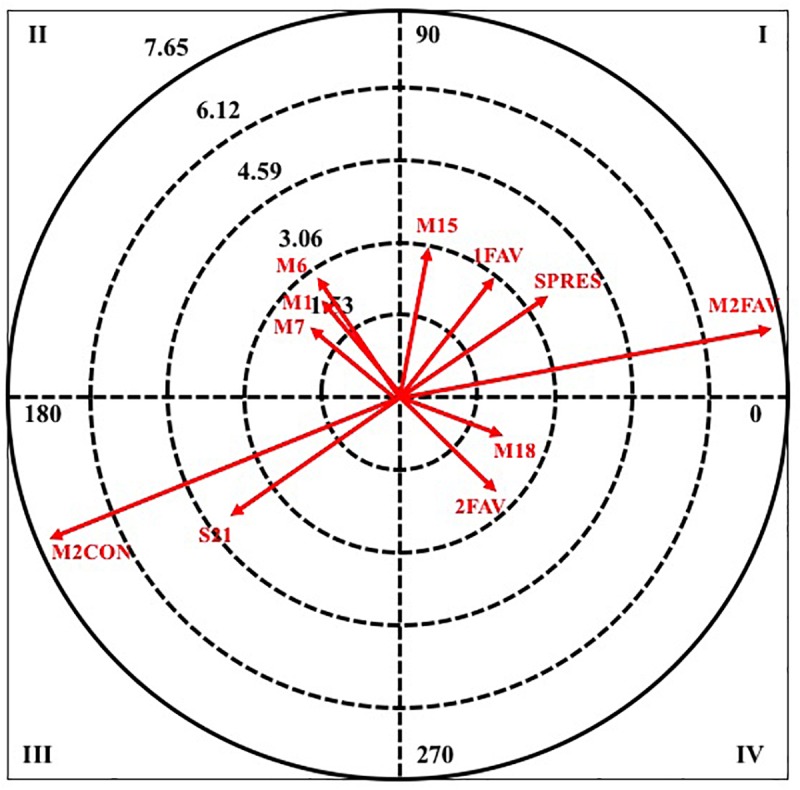
Vector maps for focal behavior GOAL2 in male category.

**FIGURE 3:**
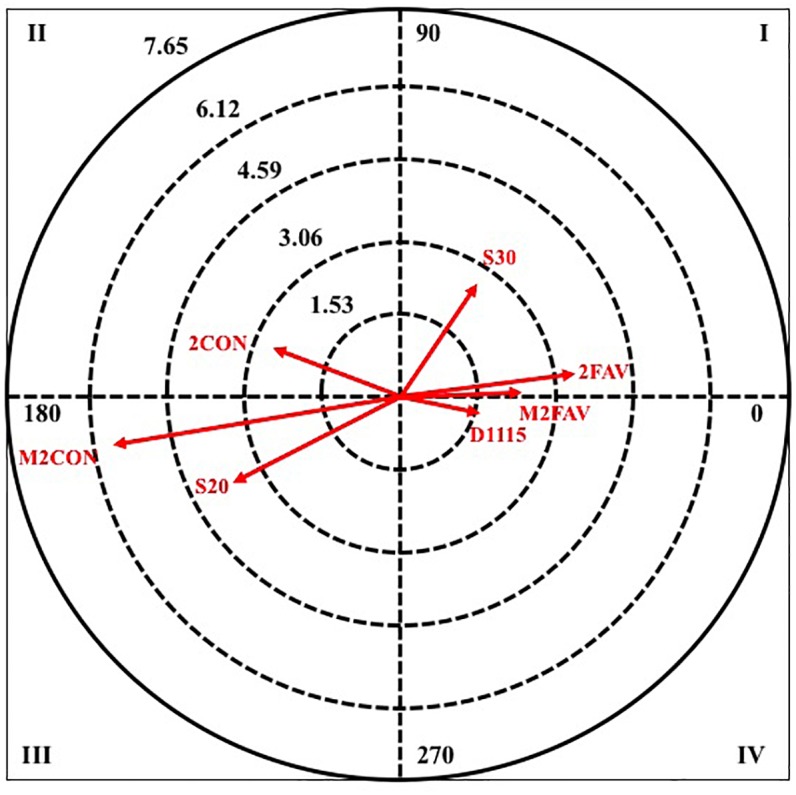
Vector maps for focal behavior GOAL2 in female category.

Next, in Figures 2, 3, it can observe the graphical representation resulting from the analysis of polar coordinates for this criterion behavior.

Table 5 shows the results obtained when the criterion behavior is TURN.

**Table 5:** Relationships between focal behavior TURN and conditional behaviors.

Criterion behavior	Q	Male			Female		
		Pairing behavior	Vector module	T.A.	Pairing behavior	Vector module	T.A.
**TURN**	I	1INF	3.22	61.80	S2M1	2.30	5.44
		M2LOS	2.51	56.22	SRETR	2.22	79.03
		M16	2.44	19.20	M7	2.10	58.36
		M18	2.31	2.45	D05	1.98	11.82
	II	M12	4.04	138.09	M2WIN	3.45	179.87
		M13	2.58	115.48	M1	2.33	169.95
		M14	2.43	117.99	M9	2.32	107.00
		M11	1.98	131.74	M20	2.16	158.66
	III	M2WIN	2.12	220.15	S30	2.38	239.58
		M20	2.08	205.62	M18	2.31	246.84
	IV	M17	3.87	329.85	M2LOS	2.47	348.82
		M9	3.30	309.83	M12	2.18	355.09
		TIE	2.53	298.28	M17	2.09	286.37
		M1	2.43	302.59		
		M10	1.97	285.94		

*Q, quadrant; T.A., transformed angle; TURN, attack ends with passing or catching error; 1INF, disadvantage of one; M2LOS, observed team is losing by more than two points; M16, minute six of second half; M18, minute eight of second half; M12, minute two of second half; M13, minute three of second half; M14, minute four of second half; M11, minute one of second half; M2WIN, observed team is winning by more than two points; M20, minute ten of second half; M17, minute seven of second half; M9, minute nine of second half; TIE, tie; S2M1, 2+1 defensive system; SRETR, individual defensive system; M7, minute seven of first half; D05, between 0 and 5 s; M1, minute one of first half; S30, 3:0 defensive system.*

In quadrant I, in the two categories there are four pairing behaviors, in the male category the one with the highest radius is a player in inferiority (1INF), while in the female category, it is the defensive system of the opposing team two plus one (S2M1).

In quadrant II, four paring behaviors also appear in both categories, for the male category all pairing behaviors refer to the criterion minute number, being the one with the greatest intensity of relation the minute two of the second half (M12); On the other hand, in the female category, the score of more than two goals in favor (M2WIN) stands out.

In quadrant III, again the number of pairing behaviors that are excited in both categories coincides, two in this case. Being those that greater radius presents the score of more than two goals in favor (M2WIN), in male category, and the defensive system of the opposite team 3: 0 (S30).

To finish this criterion behavior, in quadrant IV, in the male category there are five pairing behaviors, where four belong to the criterion minute number, being the one with the highest radius the minute seven of the second half (M17), and one to the result of the score in a tie (TIE). While, in the female category, three pairing behaviors are excited, being the score of more than two goals against (M2LOS), the most intense in their relationship.

**FIGURE 4:**
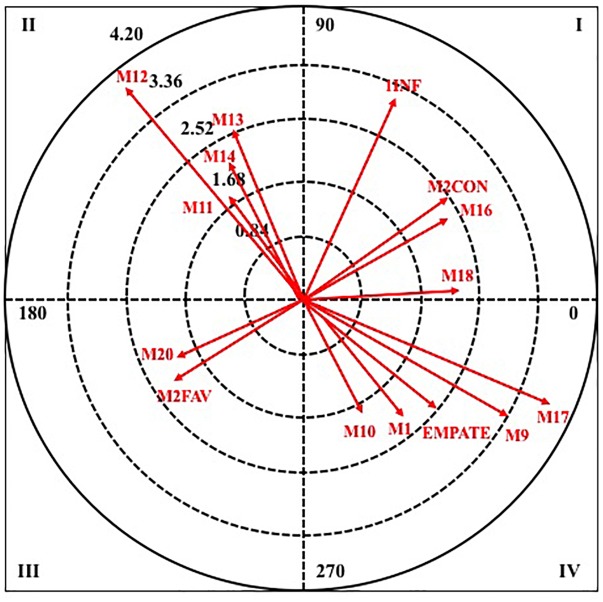
Vector maps for focal behavior TURN in male category.

**FIGURE 5:**
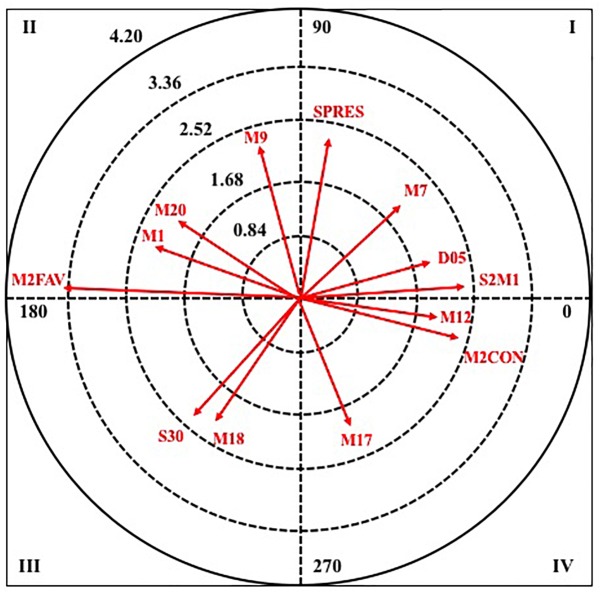
Vector maps for focal behavior TURN in female category.

Next, in Figures 4, 5, it can observe the graphical representation resulting from the analysis of polar coordinates for this criterion behavior.

Table 6 shows the results obtained when the criterion behavior is TECNF.

**Table 6:** Relationships between focal behavior TECNF and conditional behaviors.

Criterion behavior	Q	Male			Female		
		Pairing behavior	Vector module	T.A.	Pairing behavior	Vector module	T.A.
**TECNF**	I	MGOAL1	4.90	44.66	1WIN	3.68	52.66
		M2LOS	3.82	61.44	M2LOS	2.89	29.48
		M1	2.11	8.72	MGOAL2	2.52	45.22
					1SUP	2.25	6.51
	II	M18	3.07	150.00	1LOS	2.82	152.23
		M17	2.18	177.09	M2	2.30	108.96
		D610	2.01	107.88	D1620	1.97	113.80
	III	M2WIN	4.96	206.20	S2M1	2.69	240.70
					M11	2.04	183.74
	IV	M13	2.50	271.04	M6 2.15 344.52		
		2LOS	2.49	330.41	M5 2.10 339.43		

*Q, quadrant; T.A., transformed angle; TECNF, attack ends with foul; MGOAL1, golden goal of first half; M2LOS, observed team is losing by more than two points; M1, minute one of first half; M18, minute eight of second half; M17, minute seven of second half; D610, between 6 and 10 s; M2WIN, observed team is winning by more than two points; M13, minute three of second half; 2LOS, observed team is losing by two points; 1WIN, observed team is winning by one point; MGOAL2, golden goal of second half; 1SUP, advantage of one; 1LOS, observed team is losing by one point; M2, minute two of first half; D1620, between 16 and 20 s; S2M1, 2+1 defensive system; M11, minute one of second half; M6, minute six of first half; M5, minute five of first half.*

For quadrant I, the results show that in male category there are three pairing behaviors, the minute corresponding to the golden goal in the first set (MGOAL1), the score more than two goals against (M2LOS) and the minute one of the first half (M1). In female category, four behaviors appear, highlighting that it coincides with the male category, the score of more than two goals against (M2LOS).

In quadrant II, three pairing behaviors are excited in the two categories, being the minute eight of the second half (M18) in the male category, and the score of one goal against (1LOS), in the female, those with the highest radius.

In quadrant III, in male category, only a pairing behavior appears, the score of more than two goals in favor (M2WIN) and in the female category there are two, the defensive system of the opposing team two plus one (S2M1) and the minute one of the second half (M11).

Finally, in quadrant IV, two pairing behaviors are excited in both categories, in the male, the minute three of the second half (M13) and the score of two goals against (2LOS), and in the female, the minute six and five of the first half (M6 and M5).

**FIGURE 6:**
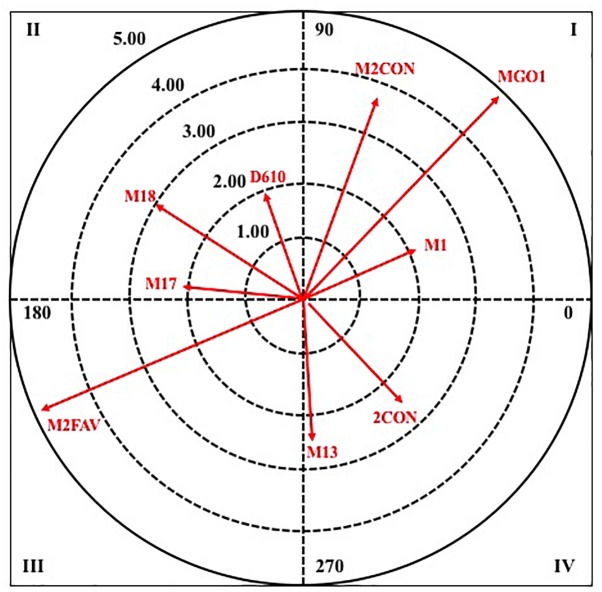
Vector maps for focal behavior TECNF in male category.

**FIGURE 7:**
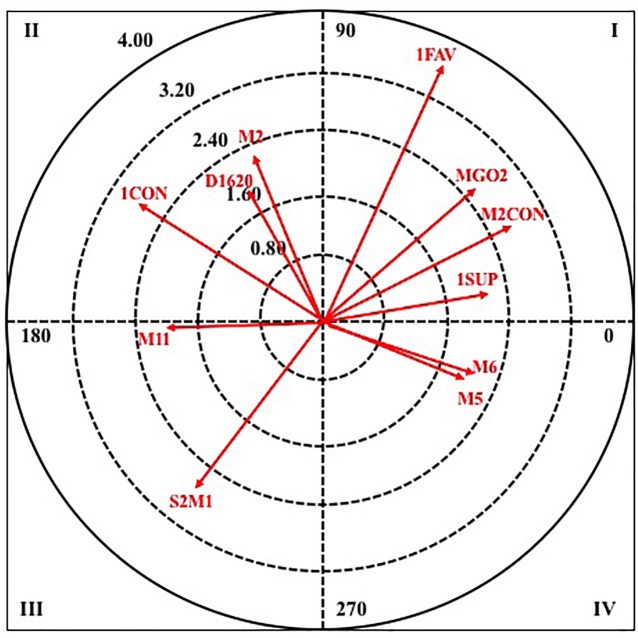
Vector maps for focal behavior TECNF in female category.

Next, in Figures 6, 7, it can observe the graphical representation resulting from the analysis of polar coordinates for this criterion behavior.

## Discussion

The objective of this research was to study the relationship of some external factors with the decisions made by beach handball players through the analysis of polar coordinates. The results obtained showed that there are differences between the flow of behaviors that are significantly related in the male category compared to the female category, this agrees with the results obtained in the investigations of Morillo-Baro et al. (2015) and Navarro et al. (2018).

In the male category, the achievement of the double value goal excites the appearance of pairing behaviors related to the result of the favorable score, as shown in quadrants one, two and four. Therefore, it can be interpreted that the success in the decision making of the players is closely related to winning. A fact that agrees with the results obtained by Conejero et al. (2017). It is important to bear this in mind, since Morillo-Baro (2011) determined that 70% of the time of the match, the score takes place with differences between plus two and minus two points; however, it must be pointed out that this is a study carried out both with a population and in a different competition. Another noteworthy result is the pressing defensive system presented by the opposing team. This can be related to the fact that, faced with open defensive systems, players are able to make appropriate decisions and show that not only the specialist is capable of making decisions, a fact that provides a great tactical wealth to the collective game in this category.

Analyzing together the other two selected criterion behaviors, it stands out that both are associated with the unfavorable score. Players commit more pass or reception errors and more technical fouls when their team is losing, which could indicate an increase in pressure to reverse that situation resulting in more precipitation. They also agree in both that they occur more in the last minutes of the matches, when the match moment is critical (overtime and last 5 min of the game when the difference of points is less than six, Navarro et al., 2012). What is in line with other works that indicated a decrease in performance and accuracy in the decision-making in the last minutes of the match (Oñoro et al., 2015). It should also be noted that errors of pass or reception also occur more when the attacking team is in numerical inferiority, a fact that agrees with that stated by Aguilar et al. (2012).

With regard to the female category, the double value goal, as it happens in the male category, is directly related to the score in favor, therefore, it can be considered that this fact favors the decisions made by the players. On the other hand, this occurs before closed defensive systems, so, there is less pressure on the player that acts as a specialist and is able to make better decisions when constructing the static attack. It should be noted that in the female category, there is a great dependence on the actions of the specialist player (Morillo-Baro et al., 2015), therefore, she bears great responsibility when she comes to making decisions. Finally, it is noteworthy that the duration of the attack is between 11 and 15 s. A fact that does not offer information about whether the decisions made by the players are modulated by this criterion, but that it agrees with the fact that it lasts half a positional attack in beach handball (Morillo-Baro, 2011) and that it is interpreted as a reasonable construction of the static attack.

With regard to the error of pass or reception, the excitement of behaviors that are related to defensive pressing systems is observed, that is, the defense acts in a more active way reducing the spaces and times of action of the attacking team, besides limiting the action of the specialist. Another interesting result is the excitement of the duration of the attack less than 5 s; this is translated into a quick attack and probably it has been little elaborated. Therefore, it can be considered that this type of defense encourages players to make quick decisions under pressure through an immediate reading of the situations that arise, carrying out a negative influence on decision-making (Ruiz et al., 2014). For that reason, it could be interpreted that in front of pressing defensive systems the female teams make fast attacks that produce a greater number of errors in the pass or reception, diminishing the effectiveness of their actions.

Finally, it is directly related to the performance of technical faults in what has been previously determined critical moment of the game, therefore, it can see again how this fact modulates in a negative way the decisions made by the players. In addition, it is curiously the excitement of behavior one in superiority, this fact can be interpreted as the teams do not handle well the different decision scenarios that are created in the game and even being in superiority are not always able to make appropriate decisions. It cannot be interpreted clearly in this conduct criterion that the result influences in some way in the decision making.

By virtue of all explained until now, one might think that in both categories a correct decision-making is linked to having a favorable result on the score. In the male category, players are able to make correct decisions against any defensive system, while in the female category when they are pressured, the decisions made are reduced properly. The numerical balance has also shown its negative influence on the decision making of the athletes, in the male category when the attacking team is in inferiority and in the female category when it is in superiority. The duration of the attack is only significantly linked to the female category, showing that when they perform elaborate attacks they make a greater number of appropriate decisions than when they perform quick attacks with little elaboration, while in the male category a decrease in the success of decision-making has been observed in the last minutes of the matches.

There is hardly any published research on this modality, which leads to be cautious when conclusions are established about the results obtained. Since the research published up to now (Morillo-Baro et al., 2015; Navarro et al., 2018) had discussed the analysis of polar coordinates but they had not dealt with the analysis of decision making, which makes it difficult to compare these results with them. Another caution to consider is the limitation that is found when interpreting the decision-making process through the observation of the behaviors, ignoring other cognitive variables that surely influence. Finally, only Spanish players formed the sample, so this research has focused on the development of the game in this country.

All these results have highlighted the significant relationship between different external factors in the decisions made by beach handball players during the game. Thus, the use of observation and the technique of polar coordinates have been useful to discriminate between specific actions and behaviors, which provides data on the decisions of athletes during the competition. For all this, it would be of great interest to continue exploring this line of research on beach handball, creating new investigations with different focal behaviors to be able to establish the different patterns of the game in both categories.

## Author Contributions

AH-M, VM-S, and RR: design of the work, acquisition, analysis, and interpretation of data for the work. JPM-B: acquisition, analysis, and interpretation of the data for the work. JAV-D: acquisition and analysis of the data for the work. All authors were involved in drafting, revising, and approving the final version of the manuscript, and also agreed to be accountable for all aspects of the work.

## Conflict of Interest Statement

The authors declare that the research was conducted in the absence of any commercial or financial relationships that could be construed as a potential conflict of interest. The handling Editor declared a past co-authorship with the authors AH-M and VM-S.
